# Chemical shift perturbation mapping of the Ubc9-CRMP2 interface identifies a pocket in CRMP2 amenable for allosteric modulation of Nav1.7 channels

**DOI:** 10.1080/19336950.2018.1491244

**Published:** 2018-08-06

**Authors:** Liberty François-Moutal, David Donald Scott, Samantha Perez-Miller, Vijay Gokhale, May Khanna, Rajesh Khanna

**Affiliations:** a Departments of Pharmacology College of Medicine, University of Arizona, Tucson, Arizona USA; b Neuroscience Graduate Interdisciplinary Program, College of Medicine, University of Arizona, Tucson, Arizona USA; c Department of Pharmacology and Toxicology, College of Pharmacy, University of Arizona, Tucson, AZ; d The Center for Innovation in Brain Sciences, The University of Arizona Health Sciences, Tucson, AZ, USA

**Keywords:** CRMP2, HSQC-NMR, Microscale thermophoresis, NaV1.7, SUMOylation, Ubc9

## Abstract

Drug discovery campaigns directly targeting the voltage-gated sodium channel NaV1.7, a highly prized target in chronic pain, have not yet been clinically successful. In a differentiated approach, we demonstrated allosteric control of trafficking and activity of NaV1.7 by prevention of SUMOylation of collapsin response mediator protein 2 (CRMP2). Spinal administration of a SUMOylation incompetent CRMP2 (CRMP2 K374A) significantly attenuated pain behavior in the spared nerve injury (SNI) model of neuropathic pain, underscoring the importance of SUMOylation of CRMP2 as a pathologic event in chronic pain. Using a rational design strategy, we identified a heptamer peptide harboring CRMP2’s SUMO motif that disrupted the CRMP2-Ubc9 interaction, inhibited CRMP2 SUMOylation, inhibited NaV1.7 membrane trafficking, and specifically inhibited NaV1.7 sodium influx in sensory neurons. Importantly, this peptide reversed nerve injury-induced thermal and mechanical hypersensitivity in the SNI model, supporting the practicality of discovering pain drugs by indirectly targeting NaV1.7 via prevention of CRMP2 SUMOylation. Here, our goal was to map the unique interface between CRMP2 and Ubc9, the E2 SUMO conjugating enzyme. Using computational and biophysical approaches, we demonstrate the enzyme/substrate nature of Ubc9/CRMP2 binding and identify hot spots on CRMP2 that may form the basis of future drug discovery campaigns disrupting the CRMP2-Ubc9 interaction to recapitulate allosteric regulation of NaV1.7 for pain relief.

## Introduction

Studies over the last fifteen years have identified the presence of several post-translational modifications (PTMs) within the axonal collapsin response mediator protein 2 (CRMP2) that act as a “molecular switchboard” to facilitate both canonical (neurite outgrowth, axon specification, tubulin binding) as well as atypical (regulation of ion channels) functions of this protein [1–6]. Among these modifications, we demonstrated that SUMOylation (addition of a small ubiquitin-like modifier (SUMO) protein) on lysine 374 of CRMP2 controlled trafficking and activity of the voltage-gated sodium channel NaV1.7 [4,7,8]. Despite a clear link to human pain syndromes [9], the potential for NaV1.7 as a drug target has not yet been realized clinically. Thus, NaV1.7 remains a prized pain target. Indirectly targeting NaV1.7 via interfering with CRMP2 SUMOylation may offer an alternative approach to novel pain therapeutics. Spinal administration of plasmid encoding CRMP2 K374A, significantly attenuated pain behavior in the spared nerve injury (SNI) model of neuropathic pain [10], further endorsing the importance of SUMOylation of CRMP2 as a pathologic event in chronic pain. Using rationale design, we recently identified a heptamer peptide containing the CRMP2 SUMOylation consensus motif (CSM), which when conjugated to the cell-penetrant domain of the HIV-1 tat protein (t-CSM), disrupted the CRMP2-Ubc9 interaction, inhibited CRMP2 SUMOylation, inhibited NaV1.7 membrane trafficking, and specifically inhibited NaV1.7 sodium influx in sensory neurons [11]. Importantly, this peptide reversed nerve injury-induced thermal and mechanical hypersensitivity in the SNI model, supporting the practicality of discovering pain drugs by indirectly targeting NaV1.7 via prevention of CRMP2 SUMOylation. Here, our goal was to map the unique interface between CRMP2 and Ubc9, the E2 SUMO conjugating enzyme, so as to set the stage for future drug discovery campaigns.

Since targeting the CRMP2-Ubc9 interaction proved to be effective in reversing neuropathic pain [11], efforts towards mapping this interface are critical. Atomic-level details of Ubc9’s interaction with a few SUMO substrate proteins have been mapped in studies using X-ray crystallography and NMR [12–16]. Within target protein(s), Ubc9 recognizes a SUMOylation motif – “Ψ-K-x-D/E” – where Ψ represents a hydrophobic residue and K is the SUMO acceptor lysine [17]. In addition to this consensus sequence, some SUMO substrate proteins, like the mammalian guanosine triphosphate (GTP)ase-activating protein (RanGAP1), exhibit a second contact surface with Ubc9, which is believed to promote higher SUMOylation efficiency [18]. We generated a computational model of the CRMP2-Ubc9-SUMO interaction [11]. We used the crystal structure of RanGAP1 with Ubc9–SUMO as a structural template [19], and replaced RanGAP1 with a CRMP2 crystal structure [20]. Based on this computational model, we predicted two major points of contact: Arginine 440 of CRMP2 to Glutamate 132 of Ubc9, and Valine 371 of CRMP2 to Alanine 131 of Ubc9. In this manuscript, we map the interaction of CRMP2 to Ubc9 using [^15^N-^1^H] heteronuclear single quantum correlation spectroscopy (HSQC) and demonstrate that several residues that we predicted were indeed perturbed by CRMP2 binding. This result was largely recapitulated using the CRMP2 SUMO decoy, i.e. the t-CSM peptide. Mutating one of the computationally predicted hot spot residues on CRMP2 – Arginine 440 to Alanine – inhibited CRMP2 binding to Ubc9.

## Results and discussion

### Structural insights from a computational model of Ubc9-CRMP2

**Figure 1.**
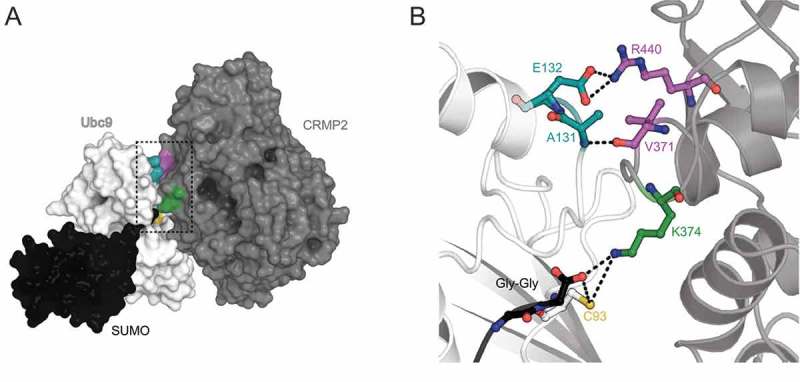
**Computational model of CRMP2-Ubc9 interaction identifies two major contact sites**. The crystal structure of RanGAP1 with Ubc9–SUMO2 (PDB: 5d2m [19]) was used as a structural template, in which RanGAP1 was substituted by CRMP2 (PDB: 2gse [20]). CRMP2 residue K374, SUMOylated by Ubc9, was used as an anchor to refine the complex using molecular minimization with the Schrodinger suite. **A**. Surface representation of the tripartite interaction between CRMP2 (*gray*), Ubc9 (*white*) and SUMO2 (*black*). **B**. Close-up view of CRMP2’s SUMOylation site (K374, *green*) interacting with the active site cysteine reside (C93, *yellow*) of Ubc9 and the SUMO2 dipeptide Gly-Gly (*black*). The modeling identified two major interactions between CRMP2 and Ubc9: (*i*) R440 of CRMP2 (purple) forms a salt bridge with E132 of Ubc9 (*turquoise*), (*ii*) V371 of CRMP2 (purple) interacts with A131 of Ubc9 (*turquoise*).

Based on the findings that CRMP2 is SUMOylated on residue Lys374 [7] and binds directly to Ubc9 with a low micromolar affinity [11], we generated a computational model of CRMP2’s interface with Ubc9 and SUMO (Figure 1). To do so, we used the structure of Ubc9 co-crystallized with both SUMO2 and the mammalian guanosine triphosphate (GTP)ase-activating protein (RanGAP1) – one the first proteins reported to be covalently linked to SUMO2 (PDB: 5d2m) [19] – as a structural template. RanGAP1 was replaced with a CRMP2 structure (PDB: 2gse [20]) and residue Lys374 was used as a docking point to refine the complex using molecular minimization with the Schrodinger suite. In this model, Lys374 from CRMP2 binds to Ubc9 active site residue Cys93 and SUMO2’s C-terminal dipeptide Gly-Gly, known to form an isopeptide bond with the amino group of substrate lysine residues [21],(Figure 1B).

This model allowed us to predict two major interactions between CRMP2 and Ubc9: (*i*) Arg440 of CRMP2 forms a salt bridge with Glu132 of Ubc9, and (*ii*) Val371 of CRMP2 forms a carbonyl-backbone amide H-bond with Ala131 of Ubc9 (Figure 1B). Reportedly, residues Asn121 to Ala131 directly interact with the SUMOylation motif of target proteins while residues Glu132-Arg141 have been shown to be important for selective target recognition [13], [18], [22]. More specifically, mutations of Ubc9 residues Gln126, Gln130, Ala131, Glu132, Tyr134, and Thr135 led to a reduction in SUMOylation of protein targets [16].

### Mapping the binding site of CRMP2 on Ubc9 using chemical shift perturbation

While *in silico* docking has been increasingly used to model protein-protein interactions, the accuracy of the prediction depends on the degree of sequence identity between the target and the template onto which it is being modelled or requires validation with experimental data (e.g. mutagenesis) on the binding interface [23,24]. In order to authenticate our CRMP2-Ubc9-SUMO model, we had to make two assumptions: (*i*) CRMP2 interacts with Ubc9 in a manner similar to that between Ubc9 and RanGAP1 and (*ii*) Lys374 of CRMP2 can act as an anchor to refine the complex. Although these assumptions are consistent with our findings of CRMP2 SUMOylation at Lys374 [7], we note that RanGAP1 and CRMP2 do not share sequence nor structural similarities, and we had no experimental data on the binding interface of CRMP2 on Ubc9.

In the present study, [^15^N-^1^H] heteronuclear single quantum correlation (HSQC) spectroscopy was used to determine which residues from Ubc9 are involved in the binding to CRMP2. In a HSQC spectrum, each amide residue yields a unique correlation peak, also called a chemical shift. Upon binding with a ligand, changes in the environment of the residues result in specific chemical shift perturbations. Usually, the affected residues are located at the interaction surface. Nevertheless, if during the complex formation, the protein undergoes significant structural modification, chemical shift perturbations can also occur in residues outside the direct interaction site. Since chemical shift perturbations are highly sensitive to binding events, it is a commonly used method to map the binding surfaces of protein-protein, protein-nucleic acid, and protein-small molecule interactions [25–27].

**Figure 2.**
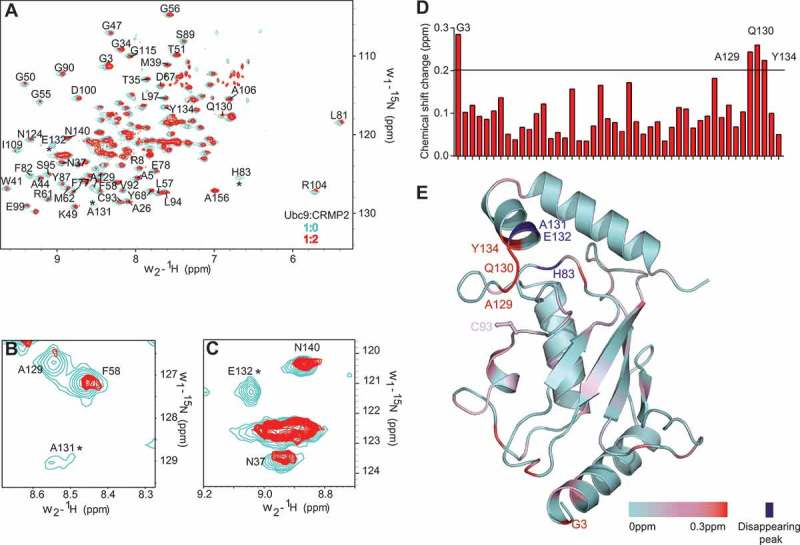
**CRMP2 binds residues from the substrate recognition site of Ubc9. A**. Superposition of [^15^N-^1^H] heteronuclear single quantum correlation spectroscopy (HSQC) spectra of [^15^N]-labeled human Ubc9 (100 µM), free and in complex with unlabeled human CRMP2. CRMP2 was incubated with Ubc9 in a CRMP2:Ubc9 ratio of 2:1. Cross-peaks: free Ubc9 (*light blue*); complex (*red*). Close-up of shifts around Ubc9 residues A129 and A131 (**B**) or E132 (**C**). Asterisks represent disappearing peaks. **D**. Average chemical shift changes for assigned residues of the [^15^N]-labeled Ubc9 upon complex formation with CRMP2. The average chemical shift changes of cross-peaks are calculated as [(5Δδ_HN_)^2^ + (Δδ_N_)^2^]^1/2^], where Δδ_HN_ represents the chemical shift change of the amide proton, and Δδ_N_ represents the chemical shift change of the amide nitrogen of an amino acid residue. **E**. Residues with chemical-shift perturbations upon CRMP2 binding plotted onto the solution structure of Ubc9 (PDB: 5d2m [19]) using a color gradient. This view is rotated approximately 90° about the vertical from Figure 1B. Abbreviations: ppm = parts per million.

The binding between CRMP2 and Ubc9 was examined using [^15^N]-labeled Ubc9 and unlabeled CRMP2. [^15^N-^1^H] HSQC spectra of Ubc9, free and in complex with CRMP2 (1:2 Ubc9:CRMP2 ratio) were compared (Figure 2A). The NMR structure of Ubc9 has been solved and the chemical shift assignments are known (Biological Magnetic Resonance Bank (BMRB) ID 4132) [28]. Most resonances of Ubc9 were not affected upon binding to CRMP2, indicating that formation of the complex does not cause overall conformational changes in Ubc9.

However, specific chemical shift perturbations were observed for several residues (Figure 2A). Notably, Ala131 and Glu132 peaks completely disappeared from the spectrum upon interaction with CRMP2 (Figure 2B and C), which denotes strong binding with the ligand [22]. Remarkably, these are the two amino acids identified in our computational model as forming direct bonds with CRMP2 (Figure 1).

A His83 peak also disappeared upon CRMP2 binding, which may indicate a local conformational change since this residue is buried in the Ubc9 structure. It was reported that the His83-Pro84-Asn85 (HPN) motif maintains the structural integrity of Ubc9’s catalytic region by maintaining the hydrogen-bonding networks and orienting the SUMO C-terminal Gly-Gly motif, required for efficient SUMO conjugation [18,22]. Moreover, mutations in the HPN motif led to a reduction of SUMOylation efficiency [29].

Some Ubc9 peaks were shifted when CRMP2 was present, we thus calculated the geometrical distance covered by each peak (Figure 2D), as described in the Methods section. Residues Gly3, Ala129, Gln130 and Tyr134 exhibited the largest chemical shift change.

To have a better picture of Ubc9’s binding site with CRMP2, we mapped the chemical-shift perturbations induced by CRMP2 interaction onto the solution crystal structure of Ubc9 (PDB: 2GRN [13]) using a color gradient (Figure 2E). While Ala129, Gln130 and Tyr134 are part of Ubc9 substrate recognition site [29], Gly3 is the only amino acid outside the active site to exhibit a significant chemical shift perturbation (Figure 2E). Another chemical shift mapping of Ubc9 with the peptide c-Jun, a transcriptional factor known to be SUMOylated, also showed such a significant chemical shift change of Gly3 upon complex formation [16]. The authors attributed this result to nonspecific interaction, since it was not reproduced with another substrate. Moreover, mutation of Gly3 into an asparagine residue, did not affect Ubc9’s affinity for RanGAP1 [30].

As CRMP2/Ubc9 binding engages residues within the substrate recognition site of Ubc9, our findings demonstrate that the CRMP2/Ubc9 complex is a substrate/enzyme type of interaction that resembles RanGAP1’s interaction with Ubc9. Furthermore, these results support our *in silico* model identifying Ala131 and Glu132 as potential “hot spots” for Ubc9 binding to CRMP2 (Figure 1).

### Recapitulating CRMP2 binding to Ubc9 using a small SUMO decoy peptide

We recently reported that that a rationally designed heptamer peptide containing the CRMP2 SUMOylation consensus motif (CSM: Gly-Lys-Met-Asp-Glu-Asn-Glu) bound Ubc9 with a similar affinity to CRMP2, efficiently disrupted CRMP2-Ubc9 interaction, and led to a reduction of SUMOylation of CRMP2 in neuronal cells [11].

**Figure 3.**
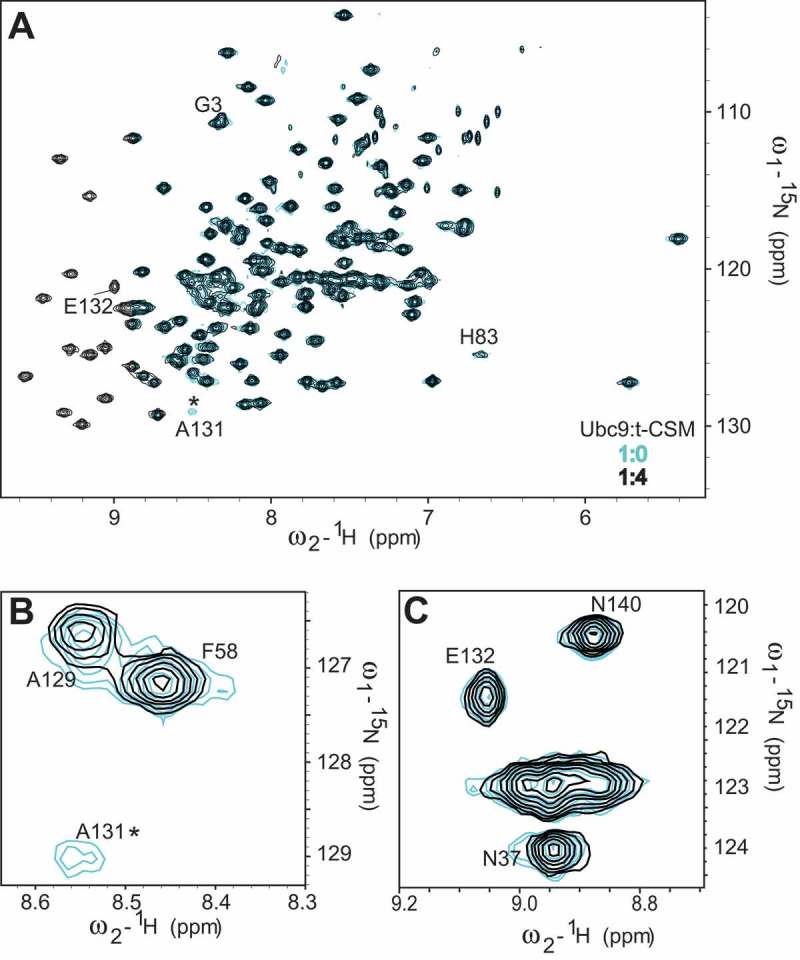
**CRMP2 SUMOylation motif (CSM) peptide binding to Ubc9 partially recapitulates the CRMP2/Ubc9 interaction**. The t-CSM peptide was incubated with Ubc9 in a t-CSM:Ubc9 ratio of 4:1. Cross-peaks: free Ubc9 (*blue*); complex (*black*). Close-up of shifts around Ubc9 residues A129 and A131 (**A**) or E132 (**B**). Asterisks represent disappearing peaks.

Thus, after identifying the Ubc9 residues involved in CRMP2 binding, we studied the chemical shift perturbations of Ubc9 in complex with the CSM peptide (Figure 3A). In a similar fashion to CRMP2 binding, t-CSM induced the disappearance of the Ala131 peak and the Ala129 peak was shifted (Figure 3B). Our computational model shows Ala131 and Ala129 to be very close to Lys374 (Figure 1) and residues Asn121 to Ala131 are known to directly interact with the SUMOylation motif of targets. In contrast, the Glu132 peak was not affected by t-CSM’s interaction with Ubc9 (Figure 3C). This was not surprising since Glu132 was predicted to interact with CRMP2 Arg440 which is not present in the CSM peptide, and Ubc9 residues Glu132-Arg141 are known to be important for selective target recognition [22].

These findings further demonstrate the ability of CRMP2 SUMOylation motif to partially recapitulate CRMP2’s binding to Ubc9.

### CRMP2 R440A mutant reduced interaction with Ubc9

**Figure 4.**
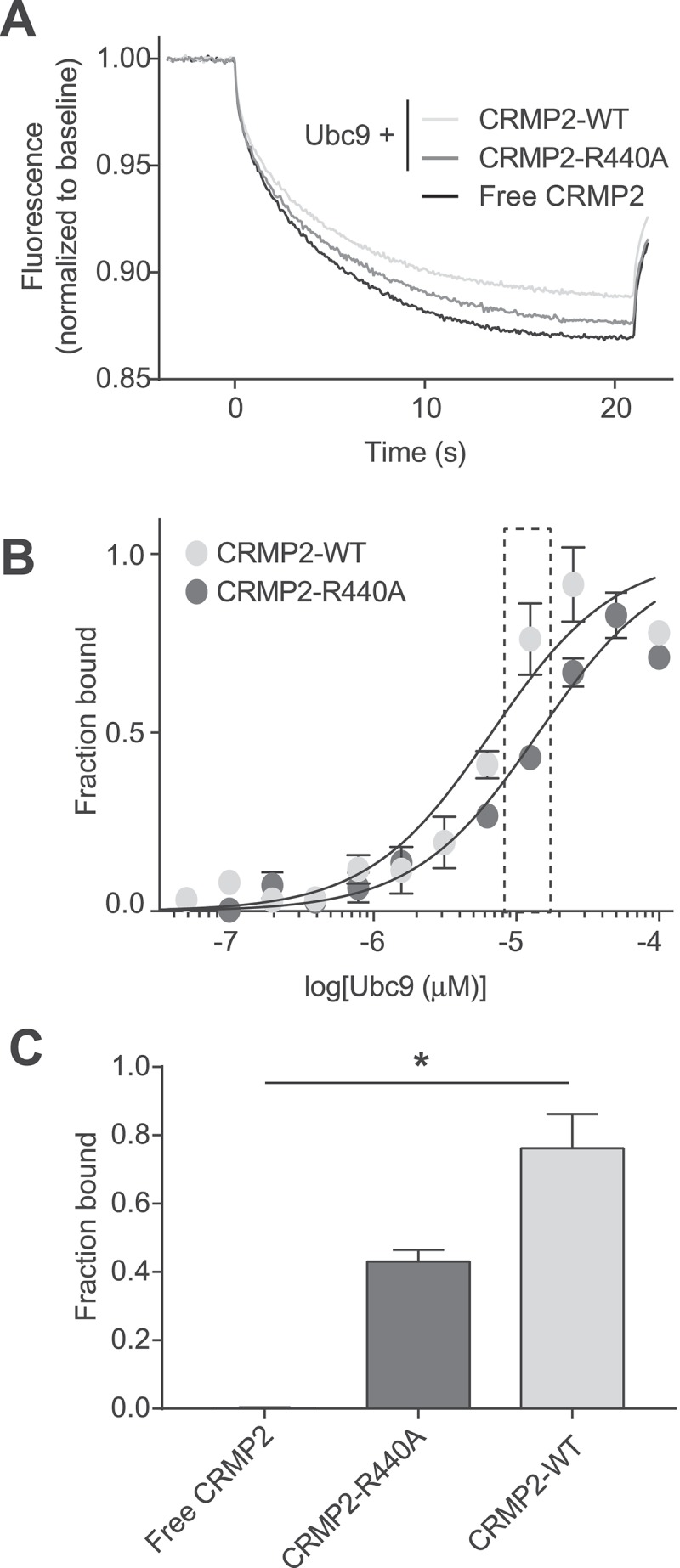
**Mutation of CRMP2 at arginine 440 reduces CRMP2-Ubc9 interaction. A**. Thermographs of Ubc9-GST (12.5 µM) with 200 nM of either NT-647-CRMP2-WT or NT-647-CRMP2-R440A. As a negative control, we used NT-647-CRMP2-WT with buffer. **B**. Binding curves were constructed between NT-647-CRMP2-WT or NT-647-CRMP2-R440A and increasing concentrations of Ubc9. At concentrations of Ubc9 between 6.25 to 25 µM, there was a significant decrease in binding (plotted as fraction of maximum) between Ubc9 and CRMP2-R440A when compared to CRMP2 WT. **C**. Quantification of CRMP2-WT or CRMP2-R440A in complex with 12.5 µM of Ubc9 (data from inset in panel **B**). Free CRMP2 in buffer is also plotted as background. Data is presented as means ± SEM (n = 3). (*, p < 0.05, one-way ANOVA with Kruskal-Wallis test).

In our computational model of CRMP2 interaction with Ubc9 (Figure 1), we identified two residues in CRMP2 that potentially form direct bonds with the Ubc9 substrate recognition site. One of those residues is Arg440 that appears to form a salt bridge with Glu132 of Ubc9. In order to validate CRMP2 Arg440 as a “hot spot’ in the binding with Ubc9, we generated a CRMP2 protein harboring an R440A mutation and we used microscale thermophoresis (MST) to study its binding to Ubc9 in comparison to wild-type CRMP2 (Figure 4).

MST measures the movement of a fluorescently labelled target protein under a temperature gradient. Upon binding of a ligand to a biomolecule, the size, charge and/or solvation properties of the biomolecule will change, resulting in a different thermodiffusion signal [31]. MST experiments showed that Ubc9 was able to significantly alter CRMP2-WT thermodiffusion, as demonstrated in our recent report ^11^, (Figure 4A,. NT647-labeled CRMP2 protein (50 nM) was incubated with varying concentrations of Ubc9 (0.003–100 μM) and apparent Kd values were obtained by fitting curves using the standard fitting model derived from law of mass action. The MST experiments revealed that Ubc9 binds to CRMP2 with an apparent K_d_ of 11.8 ± 2.0 μM. Ubc9 bound to CRMP2-R440A with an apparent K_d_ of 19.2 ± 4.9 μM (Figure 4B,. At concentrations of Ubc9 between 6.25 to 25 µM, there was a significant decrease in binding (plotted as fraction of maximum) between Ubc9 and CRMP2-R440A when compared to CRMP2 WT (Figure 4B,C). Thus, thermodiffusion of CRMP2-R440A was modified to a lesser extent by Ubc9. Quantification of the fraction of CRMP2 bound (Figure 4C), indicated that the R440A mutation reduced binding of CRMP2 to Ubc9 (at 12.5 µM) by 45% (0.43 ± 0.03 compared to 0.76 ± 0.08). This result validates residue Arg440 as a key anchor for Ubc9 binding to CRMP2.

### Conclusions

In summary, our data validate our model of CRMP2 interactions with Ubc9, where we identified Arg440 from CRMP2 and Glu132 from Ubc9 as a major point of contact for the interaction. Our results also support the expected role of Ala131 from Ubc9 in substrate recognition. Since we previously showed that targeting the Ubc9-CRMP2 interface is relevant in alleviating neuropathic pain behavior, screening for small molecules against a pocket surrounding CRMP2 Arg440 will prove useful in disrupting the CRMP2-Ubc9 interaction to recapitulate allosteric regulation of NaV1.7 to in the context of pain relief.

## Methods

### Plasmids, antibodies and other materials

Mutations to plasmid pET21-CRMP2 utilized in this study were introduced by QuikChange II XL mutagenesis kit (Cat# 200,521, Agilent, Santa Clara, CA) in the pET21-CRMP2 plasmid. Plasmids were purified from DH5α *E. coli* using the NucleoBond® Xtra Maxi kit (Cat# 740,414, Macherey-Nagel, Germany). Amplified sequences and introduced mutations were verified by DNA sequencing.

### Protein expression and purification

[Recombinant human CRMP2, CRMP2-R440A and human Ubc9-GST were expressed and purified as previously described 11,32]. Expression of recombinant human Ubc9-His, used in HSQC experiments, was induced in minimal M9 media supplemented with ^15^N-labeled ammonium chloride using 0.5 mM IPTG. Cells expressing recombinant Ubc9 were resuspended in PBS, pH 7.4, 1mM EDTA, 1 mM DTT, supplemented with Complete EDTA-free protease inhibitors (Roche, Basel, Switzerland). Disruption of the bacteria was performed by two rounds of high-pressure homogenization at 10,000 PSI with a LM10 microfluidizer (Microfluidics, Westwood), and the lysate was centrifuged 45 min at 4,500Xg at 4°C. The supernatant was loaded on a His-Trap column (GE Healthcare, Uppsala, Sweden) equilibrated with PBS, pH 7.4, 1 mM EDTA, 1 mM DTT, 1% (w/v) Triton X-100. After a washing step with TBS pH 8.0, 1 mM EDTA, 1 mM DTT, Ubc9-His was eluted with a gradient of imidazole. The fractions of interest were loaded on a HiLoad Superdex size exclusion column (GE Healthcare, Uppsala, Sweden) and eluted with 20mM Phosphate buffer pH 6.0, 500 mM NaCl, 4 mM DTT. Protein concentration was determined by a Pierce assay using bovine serum albumin as a standard. The purity of the protein was verified with SDS-PAGE. The proteins were flash frozen in liquid nitrogen and stored at −80°C.

## Hsqc-nmr

All NMR data were collected on Bruker Avance NEO 800 Mhz spectrometer with TCI-H&F/C/N probe at 25⁰C. A Transverse relaxation optimized spectroscopy (TROSY) with a solvent suppression pulse sequence was used to acquire all HSQC data. [^15^N] labelled Ubc9-His (100 µM) was incubated with either CRMP2-His (200 µM, 1:2 ratio) or with t-CSM (400 µM, 1:4 ratio).

NMR data processing and analysis was performed using programs NMRPipe [33] and Sparky (Goddard and Kneller, Sparky 3, University of California, San Francisco). Chemical shift perturbation values (Δδavg) for [^15^N] and [^1^H] nuclei were calculated using the following equation:

[(5Δδ_HN_)^2^ + (Δδ_N_)^2^]^1/2^]

where Δδ_HN_ and Δδ_H_ represent the chemical shift perturbation value of the amide nitrogen and proton, respectively. This equation was previously used to calculate Ubc9 chemical shift changes upon binding with tumor suppressor protein p53 and transcription factor c-Jun peptides, known to be Ubc9 substrates during the SUMOylation process [16].

### Microscale thermophoresis

MST, a method that monitors the thermophoretic movement of molecules in optically generated microscopic temperature gradients thereby permitting analysis of biomolecular interaction was performed as described previously [34,35]. In a typical MST-experiment the concentration of the labeled molecule is kept constant while the concentration of the unlabeled interaction partner is varied. Purified CRMP2-His wild type (WT) or R440A mutant were fluorescently labelled using the His-Tag labeling kit RED-Tris-NTA (Nanotemper, Germany) according to the manufacturer’s instructions. 50 nM of NT647-labeled CRMP2 proteins were incubated with a range of concentrations (0.003–100 μM) of Ubc9. As a negative control, CRMP2 was also incubated with Ubc9-GST’s buffer (TBS pH8.0, 1mM EDTA, 1mM DTT). The thermophoresis measurements were performed on a Monolith NT.115 (Nanotemper, Germany) using MST premium capillaries, at 40% LED and 40% MST power. Measurements were performed in triplicates. Data analysis was performed with the MO Affinity Analysis software (Nanotemper) using the Kd model (standard fitting model derived from law of mass action).
